# Regulation of Microbial Metabolic Rates Using CRISPR Interference With Expanded PAM Sequences

**DOI:** 10.3389/fmicb.2020.00282

**Published:** 2020-02-28

**Authors:** Bumjoon Kim, Hyun Ju Kim, Sang Jun Lee

**Affiliations:** Department of Systems Biotechnology, Chung-Ang University, Anseong, South Korea

**Keywords:** metabolic regulation, PAM dependence, interference, dCas9, CRISPR

## Abstract

Genome-editing CRISPR/Cas9 technology has led to the development of artificial transcriptional repressors, also known as CRISPR interference (CRISPRi). The deactivated Cas9 (dCas9) protein guided by crRNA can specifically bind to target DNA sequences, including promoters and operators, without DNA cleavage. Protospacer adjacent motif (PAM) sequence dependence may be disadvantageous in the design of target-specific CRISPRi, as the PAM sequence is essential for DNA cleavage by the CRISPR/Cas9 system. We constructed a chromosomally integrated dCas9 system (Δ*araBAD*:*dcas9*) in *Escherichia coli* under the control of the L-arabinose-inducible *P*_*BAD*_ promoter. Plasmids carrying various crRNAs with target sequences specific for the *gal* promoter (−10 region), and the *galETK* structural genes in the *gal* operon, were transformed into dCas9-expressing *E. coli*. Cellular growth and/or galactose metabolic rates were monitored in the presence or absence of gratuitous L-arabinose. D-galactose consumption and cell growth rates were partially retarded by targeting transcriptional elongation but were fully inhibited by targeting transcriptional initiation. Moreover, RT-qPCR analysis showed that CRISPRi with several modified PAM sequences can repress the transcription of target DNAs. These results indicate that cellular metabolic rates and cell growth can be controlled by targeting structural genes or regulatory regions using CRISPRi; also, a loose PAM sequence dependence can expand the DNA targets of CRISPRi.

## Introduction

The biological role of CRISPR has been identified as a prokaryotic adaptive immune system (Mojica et al., 2005; Pourcel et al., 2005). Since then, the application of CRISPR has been expanding explosively in the field of biotechnology (Doudna and Charpentier, 2014), especially because the components of CRISPR nucleases (i.e. crRNAs and Cas proteins) are separated functionally in the recognition of target nucleotide sequences and breakage of phosphodiester bonds in nucleic acids (Dagdas et al., 2017). Moreover, a specific protospacer adjacent motif (PAM) (∼3–5 bp) sequence must be located near the target sequence for the proper operation of the CRISPR/Cas system (Rath et al., 2015). In addition, the 5′-NGG sequence should be located right after target DNA sequences in the case of CRISPR/Cas9 (O’connell et al., 2014).

The CRISPR/Cas9 nuclease has been widely used in gene knockout studies in eukaryotic cells by the highly specific digestion of target DNA sequences followed by *non*-homologous end joining, resulting in the insertion or deletion of target genes (Zhang et al., 2014b). In addition, site-specific genome editing can be achieved by the introduction of single-stranded oligonucleotides and/or double-stranded DNA fragments flanking homologous regions into prokaryotic or eukaryotic cells, respectively, which can be negatively selected by the CRISPR/Cas9 nuclease (Knott and Doudna, 2018).

In addition to genome-editing tools, deactivated or dead Cas9 (dCas9) was created to be applied to artificial gene regulators (Larson et al., 2013). dCas9 has inactivated HNH and RuvC domains responsible for nuclease activity. It has been known that the target DNA sequence cannot be digested but is occupied by dCas9-crRNA complexes, which can affect the expression of target genes, and this is called CRISPR interference (CRISPRi) (Marraffini and Sontheimer, 2010a). When the dCas9-crRNA complex binds to the promoter of target genes, the initiation of transcription can be inhibited. If the complex binds to the coding region of the target gene, the elongation of transcription can be blocked (Qi et al., 2013). In prokaryotic cells, gene regulation is hardly achieved by RNA interference. Therefore, CRISPRi is very helpful in the engineering of microbial cells (Qiu et al., 2018; Xu and Qi, 2019).

Since the bacterial adaptive immune system, CRISPR, has evolved to distinguish self and *non*-self target sequences (Marraffini and Sontheimer, 2010b), the presence of the PAM sequence near the target DNA is essential for the DNA double-strand breakage generated by the Cas9-crRNA complex (Anders et al., 2014). Such PAM dependence can narrow down the possible target sequences in the Cas9-crRNA complex. However, only binding to the target is necessary for the purpose of dCas9-crRNA, which suggests that PAM dependence is lower than Cas9-crRNA dependence.

In this study, we evaluated how strongly the dCas9-crRNA complex inhibits initiation and elongation in the transcription of the *gal* operon. Also, we tested whether dCas9-crRNA can recognize DNA targets with modified PAM sequences (NNG and NGN). Our results demonstrate that cellular metabolic rates can be controlled with CRISPRi and that a loose PAM sequence dependence can expand the DNA targets of dCas9-crRNA.

## Materials and Methods

### Strains and Culture Conditions

*Escherichia coli* K-12 DH5α and MG1655 strains were used as a cloning host and a dCas9 expression host, respectively (Table 1). *E. coli* cells were grown routinely in LB medium. When needed, antibiotics such as ampicillin (50 μg/ml), kanamycin (25 μg/ml), and spectinomycin (75 μg/ml) were used.

**TABLE 1:** Strains and plasmids used in this study.

Name	Characteristics	Source/reference
**Strain**		
DH5α	*fhuA2 lac*Δ*U169 phoA glnV44* Φ*80*′ *lacZ*Δ*M15 gyrA96 recA1 relA1 endA1 thi-1 hsdR17*	Laboratory stock
MG1655	F^–^ *ilvG rfb*-50 *rph*-1	S. Adhya
HK1060	MG1655, Δ*araBAD*:*P*_*BAD*_-*dcas9*-KmR	This study
**Plasmid**		
pKD46	pSC101*ori* ^ts^, *araC*, λ *red* genes, AmpR	Datsenko and Wanner (2000)
pdCas9	P15A *ori*, *dcas9* gene, CmR	Addgene #46569
pTargetF	pBR322 *ori*, crRNA scaffold, SpR	Addgene #62226
pHK459	PAM(TGG), pBR322 *ori*, crRNA target ^498^AGGCTGTAACTGCGGGATCA^517^ in *galK*), SpR	This study
pBJ003	PAM(CGG), pBR322 *ori*, crRNA target ^573^CGAACAGAAATCACCAATGC^592^ in *galT*), SpR	This study
pBJ004	PAM(TGG), pBR322 *ori*, crRNA target ^406^TACGTTGAAAGCTTCCCGAC^425^ in *galE*), SpR	This study
pBJ005	PAM(TGG), pBR322 *ori*, crRNA target ^–30^TTCGCATCTTTGTTATGCTA^–11^ in P*_gal_*), SpR	This study
pBJ021	PAM(CAG), pBR322 *ori*, crRNA target ^503^GTAACTGCGGGATCATGGAT^522^ in *galK*), SpR	This study
pBJ022	PAM(GCG), pBR322 *ori*, crRNA target ^489^CCAGTTTGTAGGCTGTAACT^508^ in *galK*), SpR	This study
pBJ023	PAM(ATG), pBR322 *ori*, crRNA target ^497^TAGGCTGTAACTGCGGGATC^516^ in *galK*), SpR	This study
pBJ024	PAM(GGA), pBR322 *ori*, crRNA target ^499^GGCTGTAACTGCGGGATCAT^518^ in *galK*), SpR	This study
pBJ025	PAM(AGC), pBR322 *ori*, crRNA target ^504^TAACTGCGGGATCATGGATC^523^ in *galK*), SpR	This study
pBJ026	PAM(TGT), pBR322 *ori*, crRNA target ^482^CAGAAAACCAGTTTGTAGGC^501^ in *galK*), SpR	This study
pBJ027	PAM(AAG), pBR322 *ori*, crRNA target ^–1^GAAATAACCATAGCATAACA^–20^ in P*_gal_*), SpR	This study
pBJ028	PAM(GCG), pBR322 *ori*, crRNA target ^–6^AACCATAGCATAACAAAGAT^–25^ in P*_gal_*_)_, SpR	This study
pBJ029	PAM(ATG), pBR322 *ori*, crRNA target ^–31^TTTCGCATCTTTGTTATGCT^–12^ in P*_gal_*_)_, SpR	This study
pBJ030	PAM(AGA), pBR322 *ori*, crRNA target ^–2^AAATAACCATAGCATAACAA^–21^ in P*_gal_*), SpR	This study
pBJ031	PAM(TGC), pBR322 *ori*, crRNA target ^–5^TAACCATAGCATAACAAAGA^–24^ in P*_gal_*), SpR	This study
pBJ032	PAM(GGT), pBR322 *ori*, crRNA target ^–29^TCGCATCTTTGTTATGCTAT^–10^ in P*_gal_*), SpR	This study

### Genomic Integration

The primers used in this study are listed in Table 2. In order to make a *dcas9*-KmR cassette, the *dcas9* gene was amplified using plasmid pdCas9 [a gift from Luciano Marraffini, Addgene plasmid #46569 (Bikard et al., 2013)] as a template and fused with a kanamycin resistance gene by overlap PCR. The PCR products of the *dcas9*-KmR cassette with a homologous region for recombineering at the *araBAD* promoter in the chromosome were electroporated into *E. coli* K-12 MG1655 cells harboring plasmid pKD46 (Datsenko and Wanner, 2000) to generate *E. coli* HK1060 (Δ*araBAD*:*P*_*BAD*_-*dcas9*-KmR). Then, plasmid pKD46 was cured at 42°C. The disruption of the *araBAD* genes and the integration of the *dcas9* gene in HK1060 cells were confirmed by PCR.

**TABLE 2:** Primers used in this study.

Name	Sequence (5′→3′)	Description
Cas-AraC-F1	GCTTTTTATCGCAACTCTCTACTGTTTCTCCATACCCGTTTTTTTGG	Construction of *cas9*-KmR cassette
	ATGGAGTGAAACGATGGATAAGAAATACTCAATAGGCT	
KmR-AraD-R4	GTGGTGCCGGTTGCTGGAATCGACTGACCCGCCTGCGCCCAGATGGT	Construction of *cas9*-KmR cassette
	GGCGTGGCGCGAGTGTAGGCTGGAGCTGCTTCGAAGTT	
Cas-KmR-OR2	GACGGATCCCCGGAATTCAGTCACCTCCTAGCTGACTCAAA	Construction of *cas9*-KmR cassette
Cas-KmR-OF3	GCTAGGAGGTGACTGAATTCCGGGGATCCGTCGACCTGCAG	Construction of *cas9*-KmR cassette
Ara_F1	AGCAGCTCCGAATAGCGCCCTTCCCCTTGC	Confirmation of Δ*araBAD*
AraD_500dn	CCAGCCAGAAGGAGACTTCTGTCCCTTG	Confirmation of Δ*araBAD*
Sm_ATG_R	GATACTGGGCCGGCAGGCGCTCCATTGCCC	Construction of crRNA plasmids
Sm_TAA_F	GCAATGGAGCGCCTGCCGGCCCAGTATCAG	Construction of crRNA plasmids
pTarget_seq	AACGCCTGGTATCTTTATAGTCCTGTCG	Sequencing of crRNA plasmids
Cas-galK-WF	AGGCTGTAACTGCGGGATCAGTTTTAGAGCTAGAAATAGCAAG	Construction of pHK459 plasmid
Cas-galK-WR	TGATCCCGCAGTTACAGCCTACTAGTATTATACCTAGGACTG	Construction of pHK459 plasmid
*galT*_Target_F	CGAACAGAAATCACCAATGCGTTTTAGAGCTAGAAATAGCAAG	Construction of pBJ003 plasmid
*galT*_Target_R	GCATTGGTGATTTCTGTTCGACTAGTATTATACCTAGGACTG	Construction of pBJ003 plasmid
*galE* Target_F	TACGTTGAAAGCTTCCCGACGTTTTAGAGCTAGAAATAGCAAG	Construction of pBJ004 plasmid
*galE*_Target_R	GTCGGGAAGCTTTCAACGTAACTAGTATTATACCTAGGACTG	Construction of pBJ004 plasmid
P*gal* Target_F	TTCGCATCTTTGTTATGCTAGTTTTAGAGCTAGAAATAGCAAG	Construction of pBJ005 plasmid
P*gal* Target_R	TAGCATAACAAAGATGCGAAACTAGTATTATACCTAGGACTG	Construction of pBJ005 plasmid
*galK*_CAG_F	GTAACTGCGGGATCATGGATGTTTTAGAGCTAGAAATAGCAAG	Construction of pBJ021 plasmid
*galK*_CAG_R	ATCCATGATCCCGCAGTTACACTAGTATTATACCTAGGACTG	Construction of pBJ021 plasmid
*galK*_GCG_F	CCAGTTTGTAGGCTGTAACTGTTTTAGAGCTAGAAATAGCAAG	Construction of pBJ022 plasmid
*galK*_GCG_R	AGTTACAGCCTACAAACTGGACTAGTATTATACCTAGGACTG	Construction of pBJ022 plasmid
*galK*_ATG_F	TAGGCTGTAACTGCGGGATCGTTTTAGAGCTAGAAATAGCAAG	Construction of pBJ023 plasmid
*galK*_ATG_R	GATCCCGCAGTTACAGCCTAACTAGTATTATACCTAGGACTG	Construction of pBJ023 plasmid
*galK*_GGA_F	GGCTGTAACTGCGGGATCATGTTTTAGAGCTAGAAATAGCAAG	Construction of pBJ024 plasmid
*galK*_GGA_R	ATGATCCCGCAGTTACAGCCACTAGTATTATACCTAGGACTG	Construction of pBJ024 plasmid
*galK*_AGC_F	TAACTGCGGGATCATGGATCGTTTTAGAGCTAGAAATAGCAAG	Construction of pBJ025 plasmid
*galK*_AGC_R	GATCCATGATCCCGCAGTTAACTAGTATTATACCTAGGACTG	Construction of pBJ025 plasmid
*galK*_TGT_F	CAGAAAACCAGTTTGTAGGCGTTTTAGAGCTAGAAATAGCAAG	Construction of pBJ026 plasmid
*galK*_TGT_R	GCCTACAAACTGGTTTTCTGACTAGTATTATACCTAGGACTG	Construction of pBJ026 plasmid
P*gal*_AAG_F	GAAATAACCATAGCATAACAGTTTTAGAGCTAGAAATAGCAAG	Construction of pBJ027 plasmid
P*gal*_AAG_R	TGTTATGCTATGGTTATTTCACTAGTATTATACCTAGGACTG	Construction of pBJ027 plasmid
P*gal*_GCG_F	AACCATAGCATAACAAAGATGTTTTAGAGCTAGAAATAGCAAG	Construction of pBJ028 plasmid
P*gal*_GCG_R	ATCTTTGTTATGCTATGGTTACTAGTATTATACCTAGGACTG	Construction of pBJ028 plasmid
P*gal*_ATG_F	TTTCGCATCTTTGTTATGCTGTTTTAGAGCTAGAAATAGCAAG	Construction of pBJ029 plasmid
P*gal*_ATG_R	AGCATAACAAAGATGCGAAAACTAGTATTATACCTAGGACTG	Construction of pBJ029 plasmid
P*gal*_AGA_F	AAATAACCATAGCATAACAAGTTTTAGAGCTAGAAATAGCAAG	Construction of pBJ030 plasmid
P*gal*_AGA_R	TTGTTATGCTATGGTTATTTACTAGTATTATACCTAGGACTG	Construction of pBJ030 plasmid
P*gal*_TGC_F	TAACCATAGCATAACAAAGAGTTTTAGAGCTAGAAATAGCAAG	Construction of pBJ031 plasmid
P*gal*_TGC_R	TCTTTGTTATGCTATGGTTAACTAGTATTATACCTAGGACTG	Construction of pBJ031 plasmid
P*gal*_GGT_F	TCGCATCTTTGTTATGCTATGTTTTAGAGCTAGAAATAGCAAG	Construction of pBJ032 plasmid
P*gal*_GGT_R	ATAGCATAACAAAGATGCGAACTAGTATTATACCTAGGACTG	Construction of pBJ032 plasmid
*galE*-RTL	CCACCGTTTATGGCGATCAG	RT-qPCR for transcripts of *galE* gene
*galE*-RTR	GTTCCACCATCAGCTTGCTT	RT-qPCR for transcripts of *galE* gene
*galK*-RTL	GCCTCTATGCGCGATGATTT	RT-qPCR for transcripts of *galK* gene
*galK*-RTR	CACCTTTGTCGCCAATCACA	RT-qPCR for transcripts of *galK* gene
16S-RTL	CAGCAGCCGCGGTAATAC	RT-qPCR for 16S rRNA
16S-RTR	ACCAGGGTATCTAATCCTGT	RT-qPCR for 16S rRNA

### Plasmid Construction

To generate the crRNA expression plasmids, pTargetF (a gift from Sheng Yang, Addgene plasmid #62226) was used as a template to amplify 0.9 and 1.2-kb fragments with primer pairs of Cas-galK-WF and Sm_ATG_R and Sm_TAA_F and Cas-galK-WR, respectively. These two fragments were purified for isothermal assembly using Gibson Assembly^®^ Master Mix (NEB, MA, United States) to make pHK459 plasmid (Table 1). Other crRNA plasmids carrying the spectinomycin resistance gene as a selection marker were constructed by Gibson Assembly in the same manner. Target sequences recognized by crRNAs were designed in the DNA sequences (i.e. the *gal* promoter and the *galE*, *galT*, and *galK* genes) in the *gal* operon. The extended −10 region was selected as the DNA target in the *gal* promoter. For the experiments with modified PAM sequences, we constructed additional crRNA plasmids targeting the *gal* promoter and the *galK* gene. All the crRNA plasmids were confirmed by Sanger sequencing and transformed into HK1060 cells for the growth assay.

### Growth Assay

HK1060 cells carrying each crRNA plasmid were grown in LB broth containing spectinomycin at 37°C overnight as starter cultures. Next, 1.0% of cells were inoculated in 25 ml of M9 minimal medium containing sodium succinate (final 20 mM) as a carbon source and spectinomycin in 250-ml flasks. crRNAs were expressed constitutively, and gratuitous L-arabinose (final 1 mM) was added for the expression of dCas9 proteins to form the dCas9-crRNA complex in HK1060 cells carrying crRNA plasmids. To determine whether the transcription of the *gal* operon could be negatively regulated by the dCas9-crRNA complex, D-galactose (final 20 mM) was added as an additional carbon source 6 h after the beginning of flask cultures. Optical density at 600 nm was measured every 3 h to monitor cellular growth using an Ultrospec 8000 spectrophotometer (GE Healthcare, Uppsala, Sweden).

### Transcript Analysis

Expression levels of *galE* and *galK* genes were monitored using RT-qPCR. HK1060 cells carrying each crRNA plasmid were grown M9 minimal medium containing sodium succinate (final 20 mM) as a carbon source with or without L-arabinose (final 1 mM). D-galactose (final 20 mM) was added 6 h after the beginning of flask cultures. RNAs were isolated using the RNeasy^®^ Mini kit (Cat. No. 74104; Qiagen, Hilden, Germany) 3 h after the addition of D-galactose. PCR primer sequences for the target genes were designed at the Universal Probe Library Assay Design Center^1^ and listed in Table 2. Quantitative real-time PCR (RT-qPCR) reactions were carried out on a LightCycler 96 (Roche Diagnostics, Mannheim, Germany) using the RealHelix^TM^ qPCR kit (Nanohelix, South Korea). Five nanograms of each total RNA were used in RT-qPCR reactions under the following conditions: cDNA synthesis (50°C, 40 min); denaturation (95°C, 12 min); amplification for 50 cycles (95°C, 20 s; 60°C, 1 min). The raw fluorescence data were normalized against the expression level of 16S ribosomal RNA. In order to calculate the relative abundance of *galE* (374–453 base region from +1 start codon) and *galK* (895–963 base region) transcripts, mRNA levels of *galE* and *galK* genes in the presence of L-arabinose were divided by mRNA levels of the corresponding genes in the absence of L-arabinose.

### Metabolite Analysis

The concentrations of D-galactose in the culture medium were determined by high-performance liquid chromatography (HPLC, RID-10A RI monitor, Shimadzu, Japan) with an Aminex HPX-87H column (300 × 7.8 mm, Hercules, BioRad), as described previously (Kim et al., 2013). After centrifugation of the cell culture broth, the supernatant was filtered through a 0.2-μm syringe filter. The column was isocratically eluted at 47°C with a flow rate of 0.5 ml min^–1^ using 0.01 N H_2_SO_4_.

## Results

### Repression of *gal* Operon by CRISPRi

**FIGURE 1:**
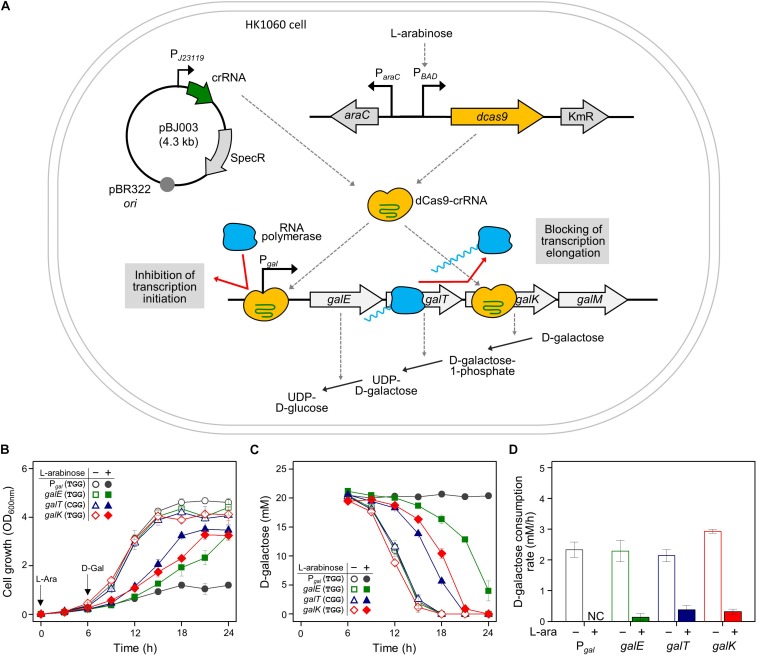
Regulation of *gal* operon and control of D-galactose metabolic rates by CRISPRi. **(A)** Expression of *gal* promoter and structural genes regulated by dCas9-crRNAs. The control of the *gal* promoter and structural *galETK* genes was used as a model system for comparing the transcriptional initiation and elongation by CRISPRi. The target DNAs in the *gal* operon can be attached by active dCas9-crRNA complexes, which are formed by dCas9 proteins expressed in the presence of *non*-metabolizable L-arabinose, and crRNAs transcribed constitutively in cells. D-galactose metabolism depends on the expression of D-galactose metabolic enzymes in the *gal* operon. **(B)** Growth profiles of HK1060 cells carrying specific crRNAs targeting *gal* promoter and structural *galETK* genes. Retarded cell growth was observed due to the repression of galactose enzymes by L-arabinose. **(C)** Change of residual D-galactose concentration in culture media. D-galactose was added at 6 h and monitored until the late stationary growth phase. **(D)** D-galactose consumption rates affected by CRISPRi. The concentration changes of residual D-galactose between 9 and 12 h were used for the calculation of D-galactose consumption rates. Arrows indicate when L-arabinose (if needed) and D-galactose were added. NC, not consumed.

Cellular growth and D-galactose utilization were monitored to assess how well CRISPRi interferes with transcription of the *gal* operon. Since CRISPRi can inhibit the initiation and elongation of transcription, the *gal* promoter and the *gal* structural genes (*galE*, *galT*, and *galK*) were used as the DNA targets of the dCas9-crRNA complex (Figure 1A). Cells carrying the L-arabinose-inducible *dcas9* gene were transformed with various crRNA plasmids. The transformed cells were grown in succinate minimal media in the presence or absence of L-arabinose, which can serve as a gratuitous inducer. Succinate was used as a carbon source to prevent carbon catabolite repression. At the early exponential phase (∼OD_600_ of 0.3), D-galactose was added to the cell culture at 6 h after inoculation. If the *gal* transcription was not inhibited by dCas9-crRNAs, the increase of cellular growth and the consumption of D-galactose could be observed.

HK1060 cells were transformed with crRNA plasmids expressing crRNAs recognizing the DNA targets with the original PAM sequence (5′-NGG). As a result, in the absence of L-arabinose, all the transformant cells reached an OD_600_ of 4, and consumed D-galactose (total 20 mM) within 18 h (Figures 1B,C). However, cell growth rates were decreased in the presence of *non*-metabolizable L-arabinose. This means L-arabinose-induced dCas9 can combine with crRNA to make dCas9-crRNA, which can interfere with the transcription of D-galactose metabolic genes.

In the case of the *gal* promoter, D-galactose was not consumed at all until the end of the experiment (Figure 1C). The *gal* promoter is composed of two overlapped extended −10 promoters (Lewis et al., 2015), which may be covered thoroughly by dCas9-crRNA complex. These results indicate that transcriptional initiation can be completely inhibited by CRISPRi.

In the case of structural genes as the inhibition targets, the cell growth and D-galactose consumption were differentially retarded. This means that the expression of *gal* genes was repressed partially by the blocking of transcriptional elongation. In the profiles of HPLC, the level of gratuitous L-arabinose was not changed in any of the samples, because the *ara* operon was disrupted by the integration of the *dcas9* gene in HK1060 cells. It was confirmed that our model CRISPRi system operated fully and partially in the promoter and structural genes of D-galactose metabolism, respectively.

### Repression of *gal* Promoter by CRISPRi With Expanded PAM Sequences

**FIGURE 2:**
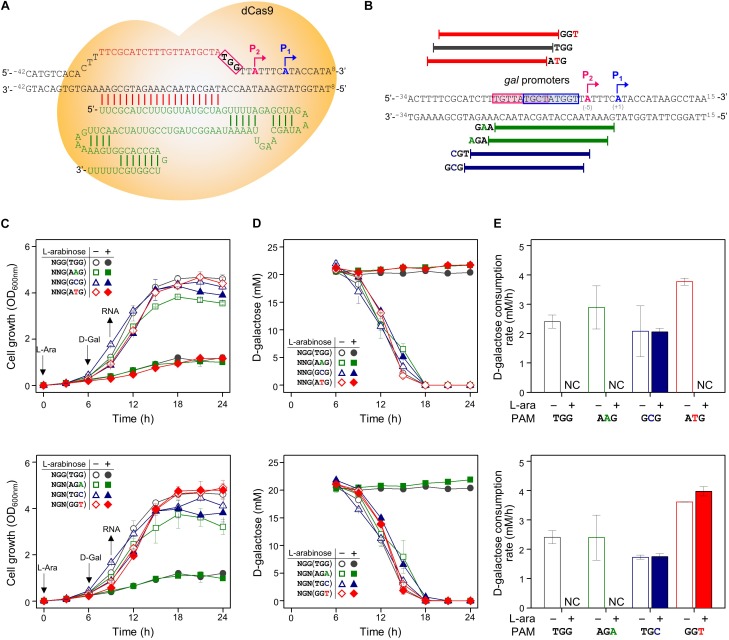
Tight repression of the *gal* promoter by CRISPRi with modified PAM sequences. **(A)** The inability of the RNA polymerase to recognize and attach to the *gal* promoter region, which is occupied by the dCas9-crRNA complex, inhibits the initiation of transcription. Blue and red boxes indicate overlapped –10 regions of *P*_1_ and *P*_2_ promoters, respectively. The *gal* promoter-specific crRNA was transcribed from plasmid pBJ005. **(B)** Design of target DNA sequences with modified PAM sequences in *gal* promoter. The *gal P*_1_ and *P*_2_ promoters overlapped each other. The modified PAM sequences of NNG (top) and NGN (bottom) were evaluated by growth profiles **(C)**, utilization of D-galactose **(D)**, and comparison of D-galactose consumption rates **(E)**. D-galactose was added at 6 h, and the concentration changes of residual D-galactose between 9 and 12 h were used to calculate D-galactose consumption rates. NC, not consumed. Arrows indicate when L-arabinose (if needed) and D-galactose were added, and when samples were taken for RNA extraction.

In order to determine whether CRISPRi follows the PAM sequence rule (5′-NGG) of CRISPR/Cas9, various crRNA plasmids were designed, as shown in Table 1. Modified PAM sequences (5′-NGN and 5′-NNG) were tested in the *gal* promoter as the DNA target of the dCas9-crRNA complex (Figures 2A,B).

**FIGURE 3:**
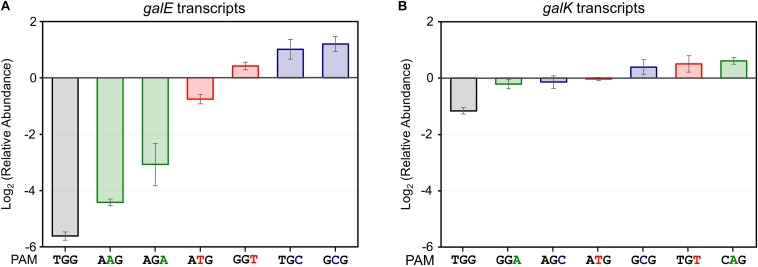
Relative transcript analysis of *galE* **(A)** and *galK* **(B)** genes affected by the presence of dCas9-crRNA complexes. *galE* and *galK* transcripts were analyzed for monitoring of transcriptional initiation and elongation, respectively. The relative abundance of *galE* and *galK* transcripts was obtained by calculating the normalized transcript data of *galE* and *galK* genes in the presence of L-arabinose (*dcas9* induced) divided by those in the absence of L-arabinose (*dcas9* uninduced).

In addition to in NGG (TGG), the growth retardation was observed in the case of NAG (AAG), NTG (ATG), and NGA (AGA) (Figure 2C). Moreover, D-galactose consumption was not observed with the expanded PAM sequences until the end of the experiments (Figure 2D). The D-galactose consumption rates (between 9 and 12 h in graphs) in the cases of NCG (GCG), NGC (TGC), and NGT (GGT) were not changed in the presence (dCas9 expressed) or absence (dCas9 not expressed) of L-arabinose (Figure 2E). Transcript analysis showed that the differential repression of the *gal* promoter by dCas9-crRNA complexes with the modified PAM sequences. The order of repression was as follows: NGG (TGG) > NAG (AAG) > NGA (AGA) > NTG (ATG) (Figure 3A). In the cases of NCG (GCG), NGC (TGC), and NGT (GGT), the repression of *gal* promoter could not be observed in the RT-qPCR experiment. These data showed that even weakly attached dCas9-crRNA complex with modified PAM sequences did not result in sufficient *gal* transcription for galactose metabolism.

### Exploration of *galK* Structural Gene as the Target of CRISPRi With Expanded PAM Sequences

**FIGURE 4:**
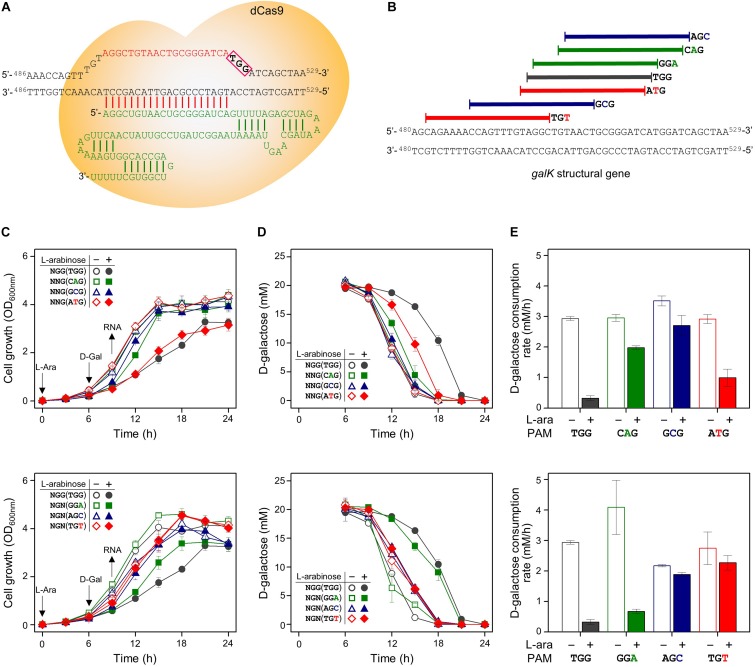
Differential repression of *galK* structural gene by dCas9-crRNAs recognizing modified PAM sequences. **(A)** Precise target recognition and proper PAM sequences are required for the blocking of transcriptional elongation in the *galK* gene by CRISPRi. The *galK*-specific crRNA was transcribed from plasmid pHK459. **(B)** Design of target DNA sequences with modified PAM sequences in *galK* gene. The modified PAM sequences of NNG (top) and NGN (bottom) were evaluated by growth profiles **(C)**, utilization of D-galactose **(D)**, and comparison of D-galactose consumption rates **(E)**. D-galactose was added at 6 h, and the concentration changes of residual D-galactose between 9 and 12 h were used to calculate D-galactose consumption rates. Arrows indicate when L-arabinose (if needed) and D-galactose were added, and when samples were taken for RNA extraction.

The same set of modified PAM sequences (5′-NGN and 5′-NNG) was tested in the *galK* structural gene as the DNA target of the dCas9-crRNA complex (Figures 4A,B). As a result, cell growth was affected slightly in the cases of NGG (TGG), NTG (ATG), and NGA (GGA) as PAM sequences (Figure 4C). According to the D-galactose consumption rate, the order of interference was as follows: NGG (TGG) > NGA (GGA) > NTG (ATG) (Figures 4D,E). The D-galactose consumption rate (between 9 and 12 h in graphs) in the case of ATG was decreased from ∼3 mM/h (dCas9 not expressed due to the absence of L-arabinose) to ∼1 mM/h (dCas9 expressed by L-arabinose).

We analyzed the abundance of mRNA of the *galK* gene in the presence or absence of dCas9-crRNA complexes (Figure 3B). The *galK* transcripts in downstream region of dCas9-crRNA complex-binding sites were monitored by RT-qPCR. In case of NGG (TGG) as a PAM sequence, the transcriptional elongation of the *galK* gene was affected by CRISPRi. However, any significant interference in the transcriptional elongation was not clearly observed in other PAM sequences using RT-qPCR analysis.

## Discussion

CRISPR interference can regulate the expression of DNA targets by inhibiting the transcription process. Designed target recognition sequences of modular crRNAs can be used to bind any DNA target to dCas9-crRNA complexes (Hawkins et al., 2015). While nonsense point mutation can affect cells permanently by deleting the function of the target gene, dCas9-mediated gene expression control allows the selection of desired time points (Quebatte and Dehio, 2017). Endogenous competing pathway genes can be repressed by CRISPRi in *E. coli* for metabolic engineering (Kim et al., 2017). Even if the genes are essential for growth, metabolic engineering with CRISPRi provides the necessary metabolites without disturbing cell growth (Cress et al., 2017).

Gene regulation using CRISPRi is carried out in two ways. First, binding of the dCas9-crRNA complex in the promoter region can fully repress transcriptional initiation. Also, elongation of transcription can be blocked by positioning the dCas9-crRNA complex in the middle of the open reading frame of the target gene (Cao et al., 2017). In our study, the *gal* operon and galactose metabolism were used as a model to demonstrate how dCas9-crRNA can precisely regulate the expression of target genes (*gal* promoter and structural *galE*, *galT*, and *galK* genes) and the corresponding metabolism (Figure 1A).

Non-metabolizable L-arabinose-induced dCas9 proteins can combine with target-specific crRNAs to make the dCas9-crRNA complex, which can bind to the DNA targets in the galactose promoter, preventing cellular consumption of D-galactose (Figure 1D). When the *gal* structural genes (*galE*, *galT*, and *galK*) were designed as the DNA targets of the dCas9-crRNA complex, we observed differential D-galactose consumption rates. RT-qPCR analysis showed that the initiation of *galE* transcription was significantly repressed, and the elongation of *galK* transcription was slightly blocked the dCas9-crRNAs with TGG as PAM sequence (Figure 3). These results might be explained by that RNA polymerase cannot bind to the *gal* promoter occupied by the dCas9-crRNA complex, but RNA polymerase can pass through some of the roadblocks of dCas9-crRNA during transcriptional elongation. Although all enzymes encoded by *galE*, *galT*, and *galK* genes are essential in galactose metabolism, the most retarded cell growth was observed in *galE-*targeted cells (Figure 1B), presumably because *galE* mutant cells cannot grow well due to the depletion of pyrimidine nucleotides (Lee et al., 2009).

The PAM sequence should be considered in CRISPR technologies when the target DNA sequence is designed. The biological role of the PAM sequence is to discriminate between *non*-self DNA from outside cells and self DNA stored in the chromosome in bacterial adaptive immune system. It has been known that the PAM sequence is indispensable for DNA cleavage by the Cas9-crRNA complex (Mojica et al., 2009). It has been reported that the hydrogen bonds between nucleotides in the PAM sequence (5′-NGG) and the two arginine residues (Arg1333 and Arg1335) in the Cas9 protein are important in DNA cleavage (Anders et al., 2014). The PAM dependence in the CRISPR/Cas9 system has been studied for expanding the target sequence and reducing off-target activity. Reportedly, the 5′-NGA sequence can function as PAM, although the modified PAM sequence exhibits less *gfp* gene cleavage activity (Zhang et al., 2014a). It is still unclear whether the PAM sequence is essential in the recognition of target sequences in CRISPR/dCas9 in bacterial cells.

The PAM dependence in CRISPRi was investigated using regulation of the *gal* promoter (Figure 2) and the *galK* gene (Figure 4) by dCas9-crRNA complex. The differential repressions of *gal* promoter by modified PAM sequences were observed by RT-qPCR analysis (Figure 3A). The order of *gal* promoter repression showed that target binding affinities of dCas9-crRNAs with modified PAM sequences might be weaker than those with the original PAM sequence (NGG). However, the CRISPRi-induced growth retardation and totally impaired D-galactose consumption suggest that NAG, NTG, and NGA can function as the original PAM sequence (NGG) in the *gal* transcription initiation (Figure 2). This means that the *gal* promoter even weakly occupied by dCas9-crRNA complex cannot be properly recognized by the RNA polymerase in the cells.

In the case of the *galK* structural gene as the DNA target of CRISPRi, transcriptional elongation can be clearly modulated by CRISPRi with the original PAM sequence (NGG) based on the results of cellular growth, metabolic rates, and transcript abundance. Since the original PAM sequence occurs frequently in the open reading frame of common genes, the expression of essential genes can be attenuated by CRISPRi with the original PAM sequence.

These results showed that the DNA target sequence for CRISPRi can be expanded, allowing the design of more specific target sequences such as narrow promoter regions. Our results also show that cellular growth and metabolic rates can be controlled by dCas9-crRNAs. The information about CRISPRi with expanded PAM sequences may be useful in the design of DNA targets and crRNAs for fine gene regulation in microbial biotechnology.

## Data Availability Statement

All datasets generated for this study are included in the article/supplementary material.

## Author Contributions

BK, HK, and SL designed the research, analyzed the data and wrote the manuscript. BK and HK performed the experiments.

## Conflict of Interest

The authors declare that the research was conducted in the absence of any commercial or financial relationships that could be construed as a potential conflict of interest.

## Footnotes

1 http://lifescience.roche.com
